# Structuring SSH within the Pasteur Network for epidemic response: setting up an African network of social scientists

**DOI:** 10.7189/jogh.15.03040

**Published:** 2025-10-24

**Authors:** Hichem Ben Hassine, Cyrine Bouabid, Chiarella Mattern

**Affiliations:** 1 Institut Pasteur de Tunis, Unité Spécialisée, Communication, Science et Société, Tunis, Tunisia; 2 Pasteur Network, Knowledge Sharing Office, Paris, France; 3 Institut Pasteur de Madagascar, Unité d'Epidémiologie et de Recherche Clinique, Équipe Santé & Sciences Sociales, Antananarivo, Madagascar

## Abstract

Addressing complex global health challenges such as epidemic preparedness, environmental impacts, and equitable healthcare access requires collaborative and interdisciplinary efforts. Social sciences and humanities (SSH) play a crucial role in understanding the historical and social contexts of disease outbreaks and in shaping strategies and guidelines informed by lessons from past epidemics. Qualitative methods, central to SSH, offer valuable insights by uncovering real-world practices, social dynamics, and power structures that often remain invisible to quantitative approaches. Effective interdisciplinarity depends on strong leadership, shared vision, clear role definitions, and dedicated time for collaboration. However, these elements are often overlooked, limiting the integration of social sciences and the development of comprehensive solutions during epidemic responses. Here we share the experience of establishing SSH within the Pasteur Network members institutions in Africa and the creation of a dedicated social science network called the African SSH Pasteur Network. We describe the process of building this network to meet growing demands for SSH expertise in health research programmes and discuss the challenges encountered and strategies employed to sustain its development.

## CONTRIBUTION TO SOCIAL SCIENCES AND HUMANITIES TO GLOBAL HEALTH RESPONSES

### Context

Addressing complex global health challenges like epidemic preparedness, environmental impacts, and access to health services requires collaborative and interdisciplinary strategies [1]. While improving population health involves integrating research and interventions across various disciplines, few studies describe or provide tools for fostering genuine interdisciplinarity [2]. Interdisciplinary research uses multiple perspectives to address complex health issues, generating evidence-based data to inform decision-makers and meet diverse population needs [1].

The crucial role of social sciences and humanities (SSH) in examining the social dimensions of health is widely recognised, with each discipline providing unique insights into different aspects of disease. Numerous reports have emphasised the need for greater contribution from SSHs, which encompass a heterogeneous group of disciplines, including sociology, anthropology, geography, international relations, and economics – each with its theoretical foundations, operational priorities, and methodologies [3,4], and all including complementary quantitative and qualitative approaches.

### Complementarity in the field of SSH

Geography, for example, will document how population movements influence the spread of pathogens [5], while psychology will analyse populations’ attitudes toward health risks [6]. More generally, the common feature of the SSHs is their methodology of data production through field research, whether quantitative, qualitative, or mixed [7].

Quantitative sciences rely on statistical methods to test hypotheses, measure variables, and generalise findings across larger groups. In contrast, qualitative sciences use methods such as interviews, focus groups, and case studies for data production. The quantitative approach offers the advantage of comparison and representativeness, but often produces data that is decontextualised. Qualitative approaches used in social sciences, meanwhile, lack representativeness, but provide contextualised data. The major strength of qualitative research is its ability to produce and analyse data within the real context experienced by participants [8]. Quantitative surveys will analyse prevalence, incidence, and risk factors, while qualitative data will reveal barriers to acceptance, root causes, and coping strategies. These two approaches are complementary and increasingly used in mixed-methods research.

Through observations and interviews, the qualitative approach offers valuable insights into these discrepancies by revealing real-world practices, social dynamics, and power structures that quantitative methods alone cannot capture. This ability to reveal hidden aspects of human behaviour demonstrates why, as Bardosh and colleagues argue [3], anthropology is most often mobilised in epidemic management, where understanding the social and cultural context is crucial for designing effective health interventions.

### SSHs and infectious diseases

Infectious diseases pose significant challenges to public health, and their spread is intricately linked to social factors. Beyond epidemiology and biology, social scientists play a crucial role in understanding, preventing, and recovering from infectious disease outbreaks [9–11]

Research on HIV has been crucial in recognising the social dimensions of health behaviours [12]. International literature highlights the importance of SSH in accumulating knowledge from past outbreaks, which informs responses to future crises. For example, lessons learned from HIV/AIDS or Ebola outbreaks have shaped responses to the COVID-19 pandemic, particularly in developing concepts such as the role of key populations [13], community engagement [14], and building trust to improve adherence to prevention measures [15]. The SSHs not only help study the history and social factors involved in a disease outbreak, but also provides strategies and guidelines drawn from previous epidemics. A study by Anoko and colleagues [14] identified 10 key lessons from the Ebola outbreak, emphasising the importance of co-constructing culturally and epidemiologically appropriate solutions, particularly through community engagement. This approach was reiterated in another study that proposed a comprehensive research protocol for generating epidemiological, sociological, and anthropological data on COVID-19 in Burkina Faso [16].

Social scientists play two key roles in supporting health professionals during epidemics. The first is research, which aims to provide a comprehensive, context-specific understanding of outbreaks. Experts are tasked with documenting various aspects of the disease, understanding health behaviours, social norms, analysing family relationships to better document the transmission chains of an infectious disease [17], analysing the interactions between healthcare providers and patients regarding treatments, and assessing public health measure compliance or acceptability among populations. The second role involves mediation between healthcare providers and local communities for trust-building in communities. For example, managing funeral practices was critical during the Ebola crisis: as traditional rituals often involve physical contact with the deceased – a significant transmission risk – anthropologists helped develop responses that balanced cultural respect with safety measures.

Currently, SSHs are often underutilised and integrated too late in health research programmes. Reticence from biomedical researchers towards social sciences, which largely stems from a lack of understanding and curiosity about different ways of ‘doing science’ [18], poses a significant challenge to efficient interdisciplinarity. Institutional constraints within organisations unaccustomed to integrating alternative perspectives further exacerbate this issue, while funding agencies often require the inclusion of social sciences in health programmes, but prioritise biomedical research, leading to an imbalance in resource allocation, often at the expense of the former.

Key conditions for effective interdisciplinarity include strong leadership to coordinate research teams and facilitate communication. Researchers must also clearly understand each other’s roles in responding to the main scientific objective. Collaborative environments should be promoted through workshops and regular result-sharing sessions to enhance cooperation and knowledge exchange. However, time for interdisciplinary dialogue and result-sharing is often overlooked during project planning. Once under way, research teams work in parallel, focussed on their own priorities and timelines, leaving little room for collaboration. As a result, dialogue is no longer a priority, hindering the co-construction of comprehensive solutions. Persistent gaps impede the full integration of social sciences in addressing epidemics [3].

This viewpoint explores the emergence of SSHs within the African Pasteur Network (PN) members and their subsequent structuring. It aims to describe the process of creating a network for social scientists within the PN member institutes, addressing the increasing demand for expertise in this field for health research programmes within the PN.

### The genesis of SSH applied to health in the PN

Several public health institutions have spearheaded initiatives and networks to integrate SSH into global health response to epidemics. Among them, there is the *Réseau Anthropologie et épidémies émergentes* [11], founded by researchers from the *Institut de Recherche pour le Développement* (*IRD*), the European Association of Social Anthropologists (EASA) [19], the Anthropological Responses to Health Emergencies [20], or the Sonar Global consortium [21]. Other initiatives exist that facilitate the collaboration between social scientists and health practitioners for emergency situations, such as the Social Science in Humanitarian Action Platform [22]

The PN is a global health alliance of 32 institutes with an extensive geographic reach, spanning 25 countries across 5 continents, fostering a dynamic community of knowledge and expertise. Although the SSH are not part of the mandates of the PN member institutes, these disciplines have gradually appeared in their research activities.

SSHs have been gaining ground within the PN in recent years, particularly in research on emerging infectious diseases, leading to their gradual integration into the scientific strategy of the PN pillars [23].

The process of integrating SSHs among the PN members in French-speaking African countries was initiated in 2010. This has been done through the development of interdisciplinary and multi-partner research projects, including a component in SSH [24–27]. It took a significant turn with the International Pasteurian Research Program in Response to Coronavirus in Africa (REPAIR) project, which involved ten African members of the PN. Similar to other interdisciplinary projects, REPAIR engaged social scientists, biologists, and epidemiologists to meet a general research objective. It created a favourable context for the development of a network in SSH for three main reasons. First, the issue of integrating social dimension was a part of the political agenda in the African countries during the COVID-19 pandemic, particularly regarding the management of prevention measures and the promotion of screening. The insights provided by social sciences into the pandemic were thus aligned with public health needs, and they helped improve the REPAIR scientific coordinators’ capacity to include social dimensions in the overall research design. Second, there was clear institutional commitment from both scientific coordinators and the hosting institution to incorporate social science components, marking a shift away from the traditional emphasis on biomedical research within the PN. This was reflected in the programme design prioritising interdisciplinarity to address the research objectives. Third, the SSH work package coordinators from the *Institut Pasteur de Madagascar* and the *Institut Pasteur de Tunis* provided the necessary scientific and strategic expertise for network development. Their commitment and experience were crucial in elevating this objective as a priority, considering their decade-long involvement in bolstering SSH capacity within their respective institutes.

Two SSH contributions were planned within REPAIR. The first included conducting a qualitative study on compliance with COVID-19 prevention measures, specifically polymerase chain reaction testing. The second contribution aimed to enhance SSH expertise among PN members in Africa by establishing a network. Below we describe the four stages of establishing this network and their timeline (**Table 1**).

**Table 1:** Timeline retracing the genesis of SSH network

Period	September to October 2021	November 2021 to January 2022	April 2022 to April 2023)	May 2023 to March 2025
**Activities**	Mapping of capacities in SSH within African PN member institutes	Selection focal points and launching the network	Capacity building, training, and sharing expertise	Maintaining the network
**Milestones**	Creation of a map of the African PN member institutes capacities and teams in SSH	Launch of the network with its members; selection of focal points each institute	Three webinars (building a SSH team in a health research center, public health and SSH, health geography); Pasteur Network course on social dimensions of epidemics (*Institut Pasteur de Madagascar*); obtaining the *ASA* project	Carrying out *ASA* project activities; collaboration with other SSH networks; extension of the network to local universities
**Anticipated next steps**	Identify key person to be part of the African SSH PN	Identify common research topics, good practices and challenges, avenues of collaboration	Planning of project activities, SHS Africa Alliance to strengthen the African SSH PN	Build on the results of *ASA* to develop new project and activities; integrate the scientific working groups of the PN

*ASA* – *Alliance SHS Afrique*, PN – Pasteur Network, SSH– Social Science and Humanities

### Network establishment phases

#### Mapping of capacities in SSH within African PN members (September to October 2021)

Between September and October 2021, a survey was conducted among the directors of ten PN members in Africa to assess the status of SSH research within their institutions. The findings, which have not been published, were shared during a workshop with REPAIR partners to validate the state of SSH research across African PN member institutes.

It resulted in three main outputs, with the first being clear institutional commitment to strengthening SSHs within their respective institutions to address public health research challenges better. Directors justified this by highlighting the significant contributions of SSH in enhancing research and the interdisciplinary perspective it offers. These include their ability to provide specific insights on analysing the social aspects of diseases, examining subtle indicators, understanding public perceptions, acquiring contextual knowledge, evaluating impacts, facilitating interventions, establishing public health policies, and improving the understanding of health-related issues. Second, the survey demonstrated the presence of SSH researchers within the African PN member institutes: out of ten institutes, seven had researchers in the field of SSH, including permanent staff, contractors, and consultants, representing a wide range of disciplines, such as sociology, anthropology, communication, educational sciences, and geography. This mapping showed that SSH teams are structured differently, depending on the research strategies of the respective institutions and laboratories, while the integration of social scientists is illustrated through dedicated SSH teams, researchers hosted in specific units, or collaborations with universities. Third, the directors also mentioned the challenges of integrating SSH into the African PN members: issues in funding and sustaining social scientist positions; lack of knowledge about these particular disciplines from other health researchers; insufficient training resources at national and international levels in applied SSHs for health (both academic and non-academic, online or in-person); and shortage of trained human resources in SSHs.

#### Identifying focal points and launching the Network (November 2021-January 2022)

Based on the survey results, we established three criteria that should govern the selection of focal points who will comprise this SSH Network:

1. Stable position: the focal point must hold a stable position (at least two years contract) in the institution to ensure the sustainability of the approach.
2. Research background: the focal point should be a researcher affiliated with the institution and possess a track record in conducting studies in the SSHs, including geography, anthropology, sociology, educational sciences, or qualitative studies.
3. Collaboration potential: if the institution has no SSH researchers, the focal point should be a member of the institute who can facilitate collaboration with local institutions and SSH researchers to explore potential partnerships.

Based on these criteria, more than thirty people were identified as ‘key persons’ in the seven African PN member institutes, to facilitate the network’s development. In institutes lacking social scientists, key individuals were identified based on their strategic positions within the local context, enabling interdisciplinary connections. These included biologists who frequently collaborate with social sciences and humanities researchers, as well as communication officers overseeing qualitative research components in research programmes.

On 21 January 2022, a kickoff workshop was organised – a milestone event for the creation of this SSH Network. This online workshop aimed to identify capacity-building needs in social science data production and analysis methodologies applied to health, while highlighting the urgency to rethink interdisciplinarity.

Workshop participants observed that health researchers frequently underestimate the unique methodologies and perspectives of social science disciplines. As a result, despite growing pressure from funders to incorporate the social dimensions of health, many research programmes continue to fall short of true interdisciplinary collaboration. This can lead to fragmented projects with limited public health impact. In some cases, biomedical researchers – perhaps unfamiliar with social science approaches – may involve social scientists primarily to meet funding criteria rather than to foster genuine cross-disciplinary engagement.

Furthermore, researchers face several challenges from multiple sources. First, comprehensibility is often an issue; disciplinary experts may struggle to understand each other due to differences in language, terminology, and conceptual frameworks, impeding effective collaboration. Second, impact asymmetry can occur, where one discipline benefits disproportionately from the collaboration, potentially compromising fairness and the long-term viability of the partnership. Finally, regarding funding, providers face challenges in developing appropriate evaluation criteria and allocating resources effectively amidst the complexity and uncertainty inherent in interdisciplinary research. Measuring impact and ensuring long-term sustainability also pose significant concerns due to fragmented expertise and the difficulty of assessing interdisciplinary outcomes [28].

Workshop participants also identified two main shared research areas: maternal and child health, and the social dimensions of epidemics. They also highlighted common data production methodologies, with a particular emphasis on qualitative social science techniques such as semi-structured interviews, focus group discussions, and mapping. Finally, many participants underscored the need to strengthen SSH teams within the institute to more effectively tackle research challenges, especially methodological ones in emergency contexts.

### Capacity building, training, and sharing expertise (April 2022 to April 2023)

While the survey demonstrated the presence of structured teams with shared expertise in analysing the social dimensions of epidemics, it also underscored the need to enhance their skills, sustain their activities, and support the training of researchers in SSH.

To address this challenge, several training activities were planned to structure this Network, designed through a co-construction process with its members. Three webinars were organised between April 2022 and January 2023 to present varying approaches to qualitative methodologies for data collection and analysis in epidemic context, share experiences in establishing SSH research teams within health institutes, and promote collaboration between SSH disciplines in health.

The most important training activity was the organisation of an international course on the social dimensions of epidemics, held in April 2023 at the *Institut Pasteur de Madagascar*. The course brought together 27 participants from 12 African countries based in several health and research institutes. It comprises 13 modules (including emergency and one health, ethics, preparedness), several workshops about qualitative methodology in epidemic context, and scientific conferences. This training, supported by the PN and the Sonar Global, Afroscreen, and TransVIHMI projects of the *IRD*, utilised educational resources developed through a collaboration between Sonar Global, TransVIHMI *IRD*, and *Réseau Anthropologie et épidémies émergentes*. It aimed to cultivate a common understanding of SSH in the context of epidemics, highlighting the importance of engaging with local stakeholders, and to meet the increasing demand for social science expertise in preparing for and responding to emerging epidemics.

### Maintaining the African SSH PN activities (May 2023 to March 2025)

**Figure 1:**
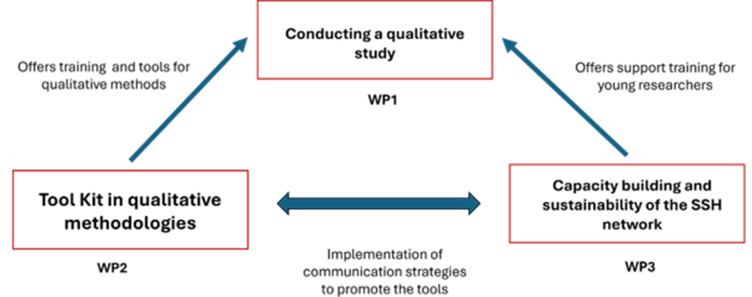
Synergy between the three main components of the *ASA* project. *ASA* – *Alliance SHS Afrique*, *SHS* – *Science Humaine et Sociale*.

The need for sustainability became paramount after the REPAIR project. Efforts focussed on maintaining and enhancing the project's impact, including sustaining the network's dynamics, fostering scientific collaborations between IPs, and enhancing the capacities of social science researchers. To build upon them, the *Alliance SHS Afrique* (*ASA*) project was launched in May 2023 as the first project within the PN entirely focussed on SSH in the context of epidemics. It involves the majority of PN's African institutes and their local scientific partners **(**Figure S1 in the **Online Supplementary Document****)**. The *ASA* aims to bolster the capacities of African health institutes to consider sociocultural dimensions in epidemics and equip them with relevant investigative tools, while seeking to enhance their ability to respond to epidemic crises by integrating SSH expertise in national and regional contexts. This project is structured around three main work packages **(****Figure 1****)**:

1. Multicenter study on vaccination strategies: this work package focusses on assessing the impact of COVID-19 on existing vaccination initiatives in the participating countries. The findings will support stakeholders in refining their national vaccination strategies using a knowledge translation approach.

2. Development of an SSH investigation toolkit: this component aims to create a comprehensive toolkit tailored for SSH research in healthcare.

3. Maintaining the activities of the African SSH PN: the final work package is dedicated to maintaining and enhancing this network of SSH experts across Africa to strengthen collaboration and knowledge exchange in the field of healthcare.

#### African SSH PN identity

The African SSH PN is the first initiative within the greater PN focussed exclusively on SSH; it has established its identity, emphasising interdisciplinary collaboration and a qualitative approach. This initiative plays a key role in advancing SSH applied to health in Africa, while also strengthening partnerships with local and regional institutions. By doing so, it aims to nurture a new generation of social scientists specialising in health in Africa, while supporting the institutionalisation and strengthening of SSH teams within the PN. Moreover, this network is instrumental in developing SSH-focussed programmes across the PN members in Africa, thereby enhancing research capabilities and promoting innovative approaches to health challenges in the region. These efforts must be sustained and scaled up through mobilising new substantial funding.

Most of the members of this network are qualitative social scientists who are convinced of their epistemic value, particularly in epidemic research. Qualitative research plays a crucial role in investigating and understanding the adaptation and implementation of policy and practice changes during a pandemic, offering in-depth insights into the dynamics of these processes. It captures diverse perspectives from healthcare professionals and patients, shedding light on their needs, concerns, and preferences, thus ensuring inclusivity in decision-making processes. Finally, qualitative research informs the development of tailored approaches to mitigate the negative impact of the pandemic on society by providing insights into how individuals and groups perceive and respond to changes, thereby enhancing the effectiveness of interventions [29].

The *ASA* project has broadened the African SSH PN’s influence, connecting with numerous institutions across and beyond Africa that collaborate with the PN members in Africa. It advances SSH applied to health in Africa by fostering strong partnerships with local and regional SSH institutions. Additionally, it aims to train a new generation of social scientists specialising in health through various educational programmes, including the massive online open course (MOOC) ‘SSH and Epidemic Management’ launched at the end of 2024, which has attracted over 2000 participants from 88 countries.

The *ASA* project has left a lasting legacy by strengthening capacities, developing research tools, and increasing the visibility of SSHs in health. Through targeted training, it has enhanced the skills of its partners and beneficiaries in key areas such as management, leadership, and the integration of gender perspectives in scientific research. In addition, the initiative has developed essential resources, including QUALILAB [30], a qualitative methodology toolkit designed to support research in epidemic contexts, particularly benefiting researchers in French-speaking African countries. The project has also contributed to advancing knowledge through studies on critical topics, such as the impact of COVID-19 vaccination on other immunisation programmes, while promoting knowledge transfer through policy notes and scientific publications. By elevating the presence of SSH at institutional, national, and international levels, *ASA* has become integrated into new research projects and contributed to the recruitment of social anthropologists in key health institutions. Ultimately, it marks a significant step toward building a healthcare response system that is both scientifically robust and socially and culturally responsive to community needs.

## CONCLUSION AND PERSPECTIVES

All the initiatives mentioned above have greatly SSHs at multiple levels – within the PN, among national policymakers and funders, and through international collaborations. They have been instrumental in integrating SSH into health research projects at the *Institut Pasteur de Tunis* and have supported the recruitment of a socio-anthropologist at the *Institut Pasteur de Dakar*. Furthermore, the *ASA* project has facilitated the establishment of a dedicated research laboratory on SSH applied to health at *Centre de Recherche Médicale et Sanitaire* (*CERMES*) and encouraged the implementation of knowledge transfer activities across various projects at the *Institut Pasteur de Tunis*. Collectively, these efforts have strengthened qualitative research methodologies, enhanced responsiveness in epidemic contexts, expanded knowledge dissemination, and promoted broader international collaboration. Despite the valuable insights gained, our African SSH PN is still facing several challenges in establishing its identity and sustaining its activities, with a key one being securing funding, mainly to maintain scientific staff. The lack of stable and substantial financial resources presents a significant obstacle to the long-term integration of SSH in health research. This situation has been exacerbated since January 2025 by an increasingly challenging funding environment, making it even harder to secure support for health research initiatives. This financial gap threatens the sustainability of SSH researcher positions, the development of SSH-integrated research programmes, and the viability of initiatives like the Alliance SHS Network. At this stage, we established a committee within our network to monitor project calls that align with our objectives, and we formed a writing team to ensure the continuity of the initiative. Even with ongoing efforts, some health researchers’ lack of awareness of SSH can lead to underestimating its importance and contribution to public health research. It is therefore crucial to emphasise the added value of SSH in understanding and managing epidemics, with SSHs integrated into research not just to satisfy funding requirements, but rather to achieve effective interdisciplinary collaboration. Beside these challenges, the shortage of qualified human resources remains problematic.

To address these issues, the network has developed a strategy to ensure its sustainability and the continuation of its activities:

- Expansion of the network: the African SSH PN plans to broaden its influence beyond the French-speaking African PN Member institutes by collaborating with additional institutions across Africa and globally. This expansion will foster new partnerships, facilitate the exchange of knowledge and best practices, and promote the integration of SSH on a wider scale.
- Training a new generation of researchers: the network is dedicated to training a new generation of SSH researchers specialising in health in Africa. The next session of the MOOC ‘SSH and Epidemic Management’ underscores this commitment, aiming to democratise access to training and enhance researchers' competencies in this critical field.
- Institutionalisation of SSH: the network is actively working towards the institutionalisation of SSH teams, ensuring their integration into the broader framework of health research initiatives. As an example, three researcher members of our network are part of the PN scientific working groups, whose task is to define the PN scientific strategy.

## Additional material


Online Supplementary Document
