# Supplementary material for: Structuring SSH within the Pasteur Network for epidemic response: setting up an African network of social scientists
Source: J Glob Health. 2025 Oct 24;15:03040. doi: 10.7189/jogh.15.03040 (PMC12548775; doi:10.7189/jogh.15.03040)
Supplement: Online Supplementary Document [file jogh-15-03040-s001.pdf]

Supplement to: Ben Hassine H, Bouabid C, Mattern C. Structuring SSH within the Pasteur Network for Epidemic Response: setting up an African network of social scientists. J Glob Health. 2025;15:03040.

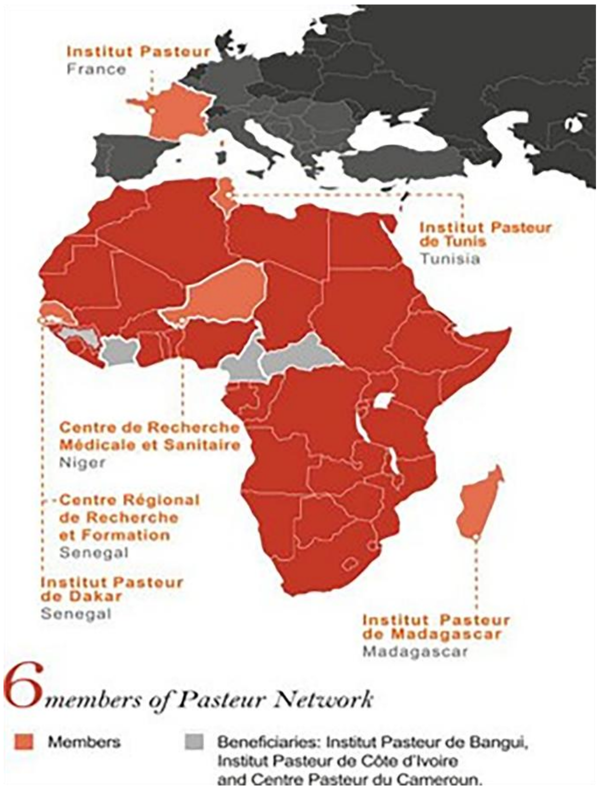

Figure S1. Alliance SHS project consortium
